# Use of the SAND balloon catheter for safe and easy laparoscopic removal of adrenal cysts

**DOI:** 10.1002/iju5.12352

**Published:** 2021-07-30

**Authors:** Jun Ito, Yasuhiro Kaiho, Hiroki Kusumoto, Yuki Kohada, Jotaro Mikami, Makoto Sato

**Affiliations:** ^1^ Department of Urology Tohoku Medical and Pharmaceutical University Sendai Miyagi Japan

**Keywords:** adrenal glands, adrenalectomy, catheter, cyst fluid, laparoscopy

## Abstract

**Introduction:**

Surgical resection should be considered for giant adrenal cysts if they are functional, if malignancy cannot be ruled out, or if there is a risk of bleeding. However, preventing cyst damage, including fluid leak, and ensuring a good field of view could be challenging in laparoscopic surgery. We report on our successful use of the SAND balloon catheter in laparoscopic adrenalectomy.

**Case presentation:**

The patient was a 40‐year‐old man with a right adrenal cyst that exhibited growth tendency. We performed laparoscopic adrenalectomy using a SAND balloon catheter through a preexisting port. Use of the catheter allowed for not only aspiration of the cyst fluid without leakage into the operative field but also gentle grasping of the cyst wall, which enabled us to easily remove the adrenal gland, including the cyst.

**Conclusion:**

Use of the SAND balloon catheter facilitates safe and easy laparoscopic resection of giant adrenal cysts.

**Abbreviation & Acronym**
CT: computed tomography

**Keynote message**

Adrenal cysts are rare but when malignancy cannot be ruled out, it would require excisions without leakage of any fluid that may be derived from the potentially malignant neoplasm and with a good operative field. Use of the SAND balloon catheter can address these issues in laparoscopic adrenalectomy.

## Introduction

Adrenal cysts are a relatively rare abnormality, but if they exhibit a tendency to grow, the possibility of malignancy and need for surgical removal should be evaluated. However, peeling these cysts from the surrounding organs and securing a good field of view in laparoscopic surgery can be difficult, particularly in cases involving giant cysts. Here, we report on the case of a patient from whom a 10‐cm adrenal cyst was safely and easily removed laparoscopically using a SAND balloon catheter, which allowed for grasping of the cyst wall without cyst fluid leakage.

## Case presentation

**Fig. 1:**
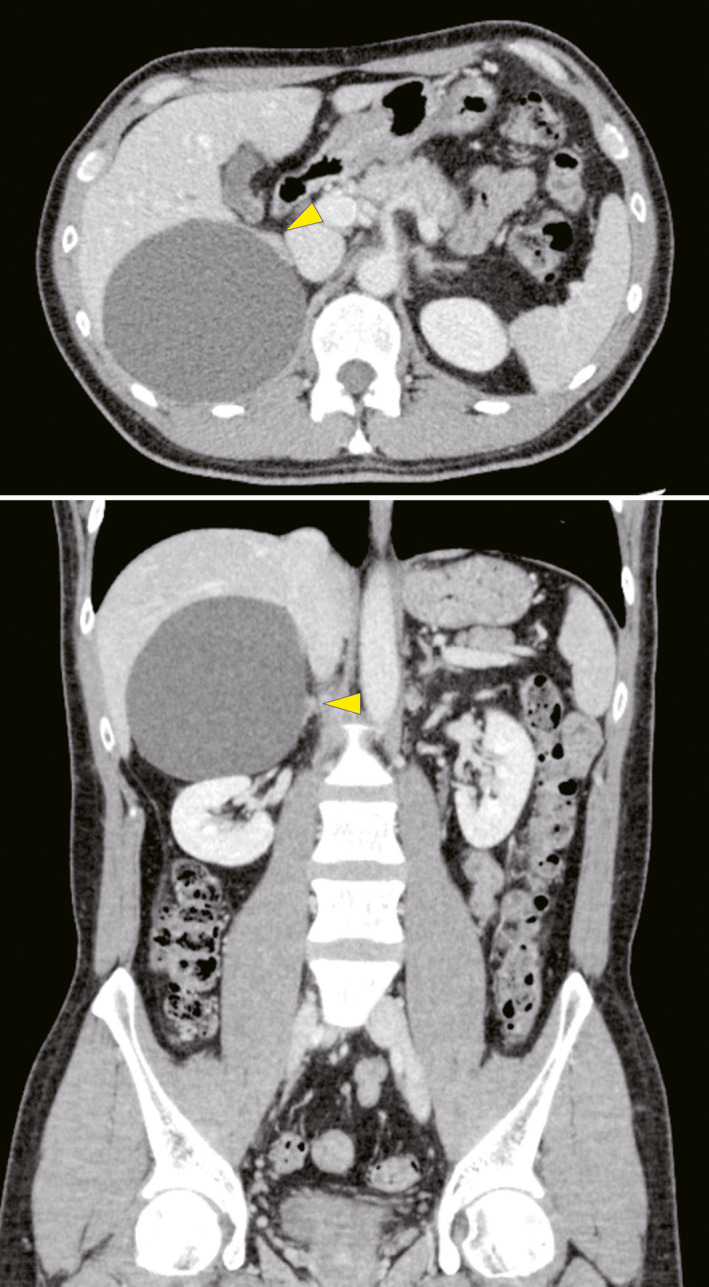
Preoperative CT image of the patient. Transverse (top) and coronal plane (bottom). A 10‐cm adrenal cyst and a normal adrenal gland (arrowhead) compressed by the cyst were seen.

The case patient was a 40‐year‐old man with a cystic lesion on his right adrenal gland, as confirmed by CT, who had been observed in our hospital for several years. We decided to remove the cyst because it exhibited a growth tendency and we could not rule out the possibility of malignancy. The cyst measured 10 cm in diameter on CT just before the surgery was performed (Fig. 1).

**Fig. 2:**
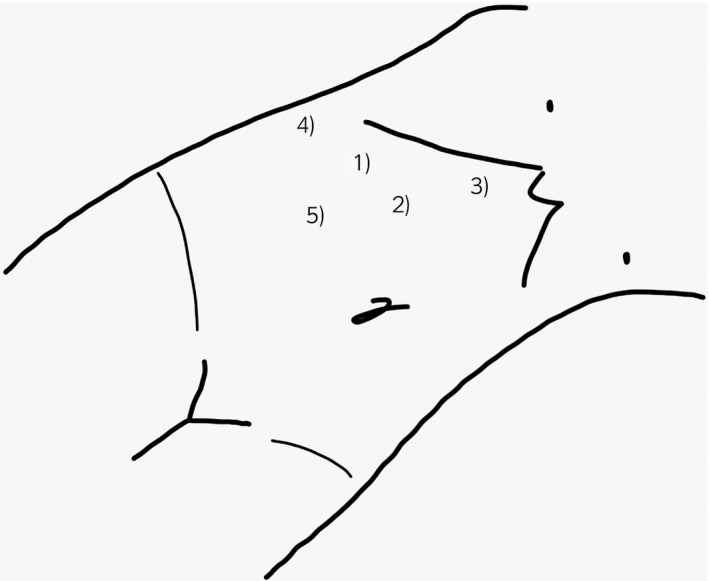
Patient and trocar positioning for a right adrenalectomy. (1) 5‐mm port for operator, (2) endoscope port, (3) 12‐mm port for operator, (4) and (5) 5‐mm ports for assistant.

The patient was placed in the left lateral position. Figure 2 shows the positioning of the ports intended for an intraperitoneal approach. First, a 20‐mm transverse incision was made below the right costal margin on the midclavicular line; the endoscopic port was placed on this incision. Subsequently, other ports were placed with the use of an endoscope: 12‐ and 5‐mm surgeon ports on the line parallel with the border of the rib arch and two 5‐mm assistant ports on the superior part of the iliac crest and the right side of the umbilicus.

**Fig. 3:**
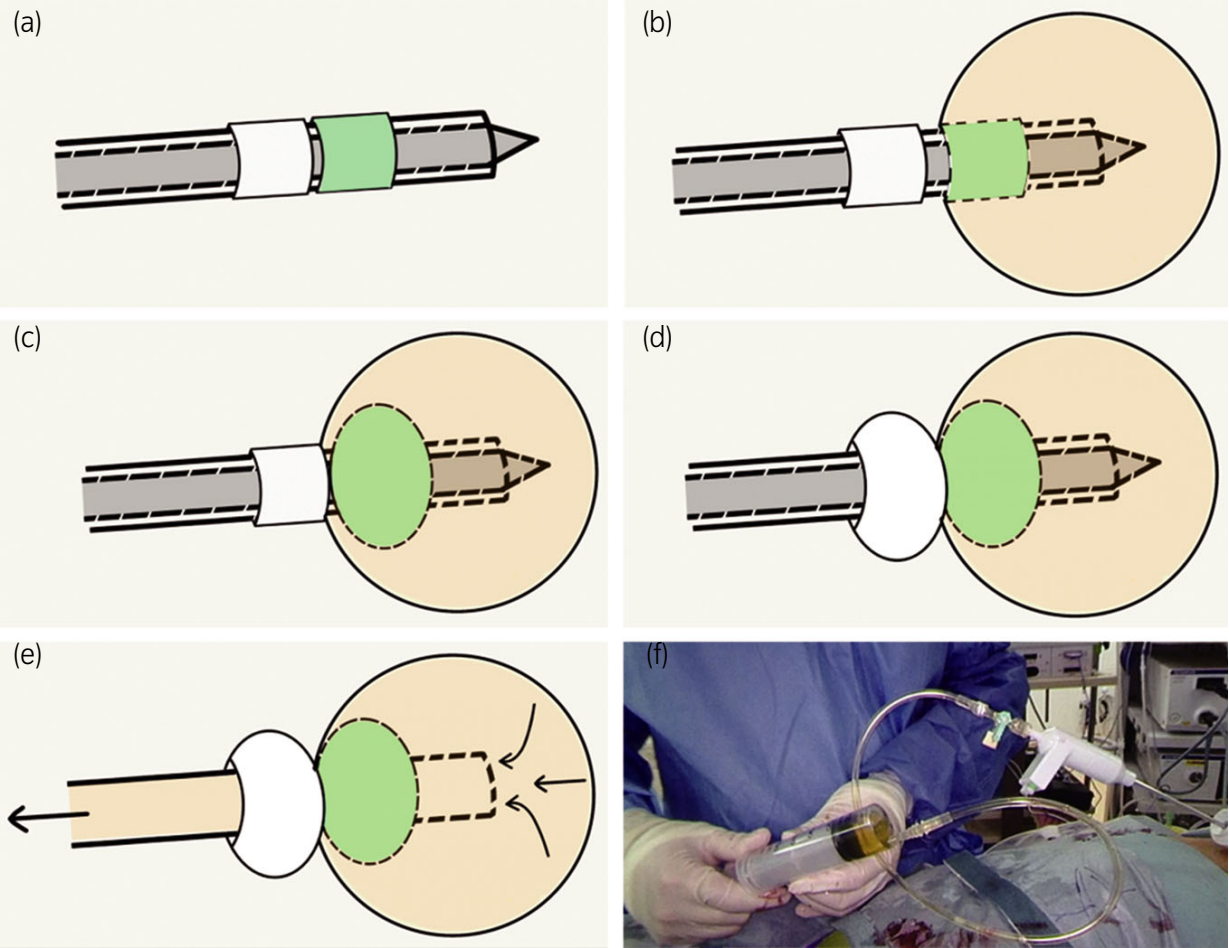
Illustration of the SAND balloon catheter and details on the procedure for its application. (a) Composition of the SAND balloon catheter: an outer cylinder with two balloons (proximal balloon and distal balloon) and an inner needle. (b) First, the cyst wall should be punctured with the inner needle until the distal balloon completely enters the cyst. (c) Next, the distal balloon should be inflated to prevent leakage of the cyst fluid. (d) Subsequently, the proximal balloon should be inflated to allow the balloons to grasp the cyst wall firmly without damage. (e) Finally, the inner needle should be removed to drain the cyst fluid. (f) The discharged fluid can be externally collected (i.e., outside the body).

**Fig. 4:**
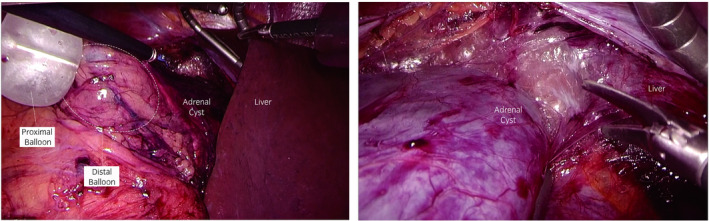
Left: The SAND balloon catheter was punctured into the cyst and pulled out caudally after cyst fluid had been drained. Right: The view of the detached surface from the liver on the cranial side of the cyst became clearer.

After performing a Kocher maneuver to expose the inferior vena cava, we incised the peritoneum from the caudal side of the liver through the surface of the inferior vena cava to the caudal edge of the cyst. Lifting the liver and upper pole of the kidney allowed us to identify the central adrenal vein and to control it with metal clips. Detaching the cyst from the psoas muscle and the surface of the liver revealed that we could not observe the cranial aspect of the cyst because of the cyst itself. To address this difficult situation, we inserted a SAND balloon catheter (Hakko Shoji, Tokyo, Japan) through an assistant port. After puncturing the cyst (Fig. S1) and aspirating some inner fluid through the inner needle of the SAND balloon catheter to prevent overflow, we inflated the distal balloon inside the cyst and the proximal balloon outside the cyst in sequence to hold the cyst wall with these balloons. Removing the inner needle led to complete discharge of the inner fluid of the cyst (Fig. 3). This procedure allowed us not only to externally collect the fluid from the cyst, for which malignancy had not been ruled out, without any leakage to the operative field but also to pull out the cyst so as to easily perform adhesiolysis with a good field of view (Fig. 4). Finally, the adrenal gland, including the cyst, was placed in a retrieval bag and extracted from the 20‐mm endoscopic port incision without additional excision. The operating time was 220 min, and the calculated blood loss was 10 mL. The pathological finding showed lymphangiomatous endothelial cyst without malignant features.

## Discussion

Cystic lesions in the adrenal gland are relatively rare and have been reported to be occasionally found incidentally during physical examinations and evaluations of other diseases.^1^, ^2^ Some studies have shown that 4–17% of adrenal cysts may be caused by malignancies of the adrenal gland,^1^, ^3^, ^4^, ^5^ and others have indicated that distinguishing between benign and malignant lesions based only on imaging methods, such as CT, can be difficult.^3^, ^4^, ^6^, ^7^ Therefore, surgical resection, from which an accurate pathological diagnosis could be obtained, should be considered for cysts with bleeding or degeneration that resembles coagulative tumor necrosis,^4^ as well as for functional adrenal cysts and cysts larger than 5 cm, which cause an increased risk of bleeding.^8^

The SAND balloon catheter was developed for laparoscopic resection of ovarian cysts. The SAND balloon is 300 mm long, 5.5 mm in diameter and can be inserted through the laparoscopic port. The price per balloon is about 150 dollars. It has been used in other laparoscopic procedures, including cholecystectomy and surgery for congenital hydronephrosis.^9^, ^10^, ^11^ To the best of our knowledge, this is the first report to describe use of the SAND balloon catheter to treat an adrenal cyst. Laparoscopic surgery for adrenal glands is preferred over open surgery because of the lower postoperative pain, fewer wound‐related complications, and better cosmetic considerations associated with it.^8^, ^12^ Although laparoscopic surgery for obviously invasive adrenal carcinoma is contraindicated, the indication for adrenal glands suspected of containing cancer cells or with large adrenal cysts is still unclear owing to the small number of cases reported. In recent years, cases in which laparoscopic surgery is selected for adrenal glands with large cysts, such as benign adrenal cysts and pheochromocytoma with large cysts, have been described.^13^, ^14^ When laparoscopic surgery is performed for a giant cyst, such as that in our case patient, ensuring a good field of view by grasping the adrenal cyst without inner fluid leakage can be challenging but is necessary. The SAND balloon catheter solves this most difficult part of the surgery. In the procedure we performed, after the cyst wall was punctured with the inner needle of the SAND balloon catheter, the distal balloon was inflated to prevent leakage of the remaining fluid in the cyst into the body. The proximal balloon was then inflated, allowing the two balloons to grasp the cyst wall firmly without damage. Furthermore, use of the SAND balloon catheter not only led to shrinking of the cyst, thus opening the surgical field of view, but also made pulling out the cyst and peeling off its adhesion easier.

In conclusion, use of the SAND balloon catheter in laparoscopic adrenalectomy for giant adrenal cysts is viable. Adrenal cysts that safely suck the internal solution out of the body through a SAND balloon catheter could be retracted in various directions, and the subsequent peeling operation could be performed easily and safely even with a limited surgical field of view.

## Conflict of interest

The authors declare no conflicts of interest.

## Approval of the research protocol by an Institutional Reviewer Board

This article is not a case series, which does not require an approval of the research protocol by an institutional reviewer board.

## Informed consent

Written informed consent for publication of this case report was obtained from the patient.

## Supporting information


**Fig. S1.** The intraoperative gross findings of the adrenal cyst and the normal adrenal gland. It was clear that the normal adrenal gland was compressed by the cyst and was present at the limbus, so it was possible to puncture the cyst avoiding the normal adrenal gland without intraoperative ultrasound sonography.

## References

[1] Sebastiano C , Zhao X , Deng FM , Das K . Cystic lesions of the adrenal gland: our experience over the last 20 years. Hum. Pathol. 2013; 44: 1797–803. 23618356 10.1016/j.humpath.2013.02.002

[2] Neri LM , Nance FC . Management of adrenal cysts. Am. Sur. 1999; 65: 151–63. 9926751

[3] Tiberio GA , Bonardelli S , Baiocchi GL *et al*. Cystic type adrenal mass. Clinical‐radiologic contribution to 7 cases treated with surgery. Chir. Ital. 2003; 55: 681–6. 14587112

[4] Erickson LA , Lloyd RV , Hartman R , Thompson G . Cystic adrenal neoplasms. Cancer 2004; 101: 1537–44. 15378490 10.1002/cncr.20555

[5] Chien HP , Chang YS , Hsu PS *et al*. Adrenal cystic lesions: a clinicopathological analysis of 25 cases with proposed histogenesis and review of the literature. Endocr. Pathol. 2008; 19: 274–81. 18972224 10.1007/s12022-008-9046-y

[6] Passoni S , Regusci L , Peloni G , Brenna M , Fasolini F . A giant adrenal pseudocyst mimicking an adrenal cancer: case report and review of the literature. Urol. Int. 2013; 91: 245–8. 23548497 10.1159/000346754

[7] Mohan H , Aggarwal R , Tahlan A , Bawa AS , Ahluwalia M . Giant adrenal pseudocyst mimicking a malignant lesion. Can. J. Surg. 2003; 46: 474. 14680359 PMC3211765

[8] Wedmid A , Palese M . Diagnosis and treatment of the adrenal cyst. Curr. Urol. Rep. 2010; 11: 44–50. 20425637 10.1007/s11934-009-0080-1

[9] Vizza E , Cutillo G , Patrizi L , Saltari M , Baiocco E , Corrado G . Use of SAND balloon catheter for laparoscopic management of extremely large ovarian cysts. J. Minim. Invasive Gynecol. 2011; 18: 779–84. 21802377 10.1016/j.jmig.2011.06.019

[10] Ikegami T , Shirabe K , Yoshizumi T , Kayashima H , Maehara Y . Use of the SAND balloon catheter in single‐incision laparoscopic cholecystectomy for acute cholecystitis. Asian J. Endosc. Surg. 2013; 6: 134–6. 23601999 10.1111/ases.12005

[11] Nozaki T , Asao Y , Ito T *et al*. Retroperitoneoscopic nephrectomy for symptomatic hydronephrosis using a SAND balloon catheter. J. Laparoendosc. Adv. Surg. Tech. A 2011; 21: 629–33. 21745100 10.1089/lap.2010.0295

[12] Winfield HN , Hamilton BD , Bravo EL , Novick AC . Laparoscopic adrenalectomy: the preferred choice? A comparison to open adrenalectomy. J. Urol. 1998; 160: 325–9. 9679870 10.1016/s0022-5347(01)62884-2

[13] Mishra AK , Agarwal G , Agarwal A , Mishra SK . Laparoscopic adrenalectomy of large cystic pheochromocytoma. Surg. Endosc. 2001; 15: 220. 11285972 10.1007/s004640040036

[14] Klimopoulos S , Perdikides T , Fratzidou E , Pissiotis CA . Laparoscopic resection of a large right adrenal gland cyst. Surg. Endosc. 1995; 9: 1295–7. 8629213 10.1007/BF00190163

