# Supplementary figures and images for: Use of the SAND balloon catheter for safe and easy laparoscopic removal of adrenal cysts
Source: IJU Case Rep. 2021 Jul 30;4(6):371–4. doi: 10.1002/iju5.12352 (PMC8560453; doi:10.1002/iju5.12352)

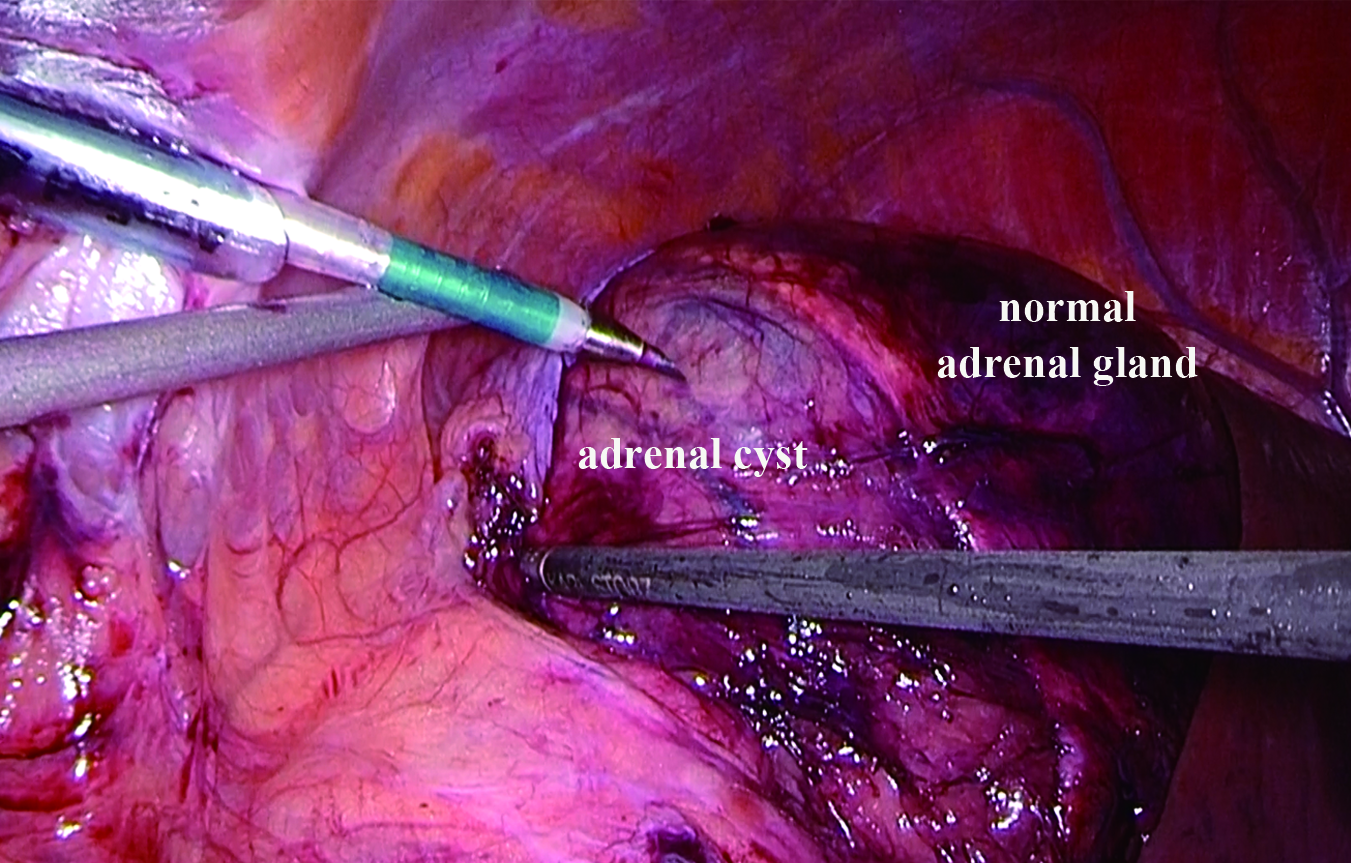

Supplement: Supplementary file 1 — Fig. S1. The intraoperative gross findings of the adrenal cyst and the normal adrenal gland. It was clear that the normal adrenal gland was compressed by the cyst and was present at the limbus, so it was possible to puncture the cyst avoiding the normal adrenal gland without intraoperative ultrasound sonography. [file IJU5-4-371-s001.tif]
